# Marginal bone loss in single implant-retained mandibular overdentures using two different healing protocols and two different types of attachments: A 2-year follow-up of a randomized controlled trial

**DOI:** 10.1007/s00784-025-06617-6

**Published:** 2025-12-05

**Authors:** Marwa Abdel Aal, Matthias Kern, Suzy N.Naiem, Iman Abdel wahab Radi, Nouran Abdel Nabi, Amr Ahmed Naguib, Mohamed Sad Chaar

**Affiliations:** 1 Department of Prosthodontics, Faculty of Dentistry, Cairo-University, Cairo, Egypt; 2 Department of Prosthodontics, Christian Albrecht University of Kiel, University Hospital Schleswig-Holstein, Campus Kiel, Germany; 3 Department of Periodontology, Faculty of Dentistry, Cairo University, Cairo, Egypt; 4 Vice Dean School of Dentistry, Badya University, Cairo, Egypt

**Keywords:** Marginal bone loss, Mandibular midline implant, Implant overdenture, Submerged, Non-submerged, Ball attachment, CM-LOC attachment

## Abstract

**Objectives:**

**“**The objective of this randomized control trial is to compare the marginal bone loss (MBL) in single implant-retained mandibular overdentures (SIRMO) retained by two different types of attachments using two healing protocols after a 2-year follow-up.

**Materials & methods:**

SIRMOs were constructed for eighty completely edentulous patients. The latters were randomized based on the healing protocol and the used attachments into 4 equal groups: Submerged healing with ball attachment (BS), submerged with CM-LOC attachment (CS), non-submerged with ball attachment (BN), and non-submerged with CM-LOC (CN). Standardized peri-apical radiographs at baseline, 12 and 24 months were recorded to measure MBL. Comparisons for numerical data were performed using either Mann Whitney-U or Kruskal Wallis tests. Binary and categorical data were compared using chi square test. Significance level was set at *p* < .05.

**Results:**

During the healing period, 6 implants failed, and 4 drop-outs were recorded. At the second year, 27/34 ball groups and 28/36 CM-LOC groups were recalled, no significant difference between B (0.28 ± 0.44) and C (0.38 ± 0.46) favoring B group (*p* = .3), and N (0.27 ± 0.39) and S (0.43 ± 0.51) favoring N group (*P* = .6). MBL revealed no significant difference neither between B and C groups nor between N and S groups.

**Conclusion:**

Both attachments showed physiologically accepted limits of MBL, favoring the non-submerged healing protocol after a 2-year follow-up period.

**Clinical relevance:**

Submerged (S) and Non-submerged (N) protocols could be used for a SIMOR, favoring the N protocol after a 2-year follow-up.

Trial registration number: PACTR20183003085193.

## Introduction

Two interforaminal dental implants in the edentulous mandible have become a standard treatment option for retaining mandibular overdentures [1]. The single implant-retained mandibular overdenture (SIRMO) is a cost-effective treatment option with promising mid- to long-term survival rates [2–11]. Immediate loading of a single implant in the edentulous mandible revealed inferior survival than that of delayed loading and therefore it should only be considered only in exceptional cases [12].

The attachment used to retain implant overdentures might, however, play an important role in improving the overall patient satisfaction and quality of life [13, 14]. Several studies reported that using ball or locator attachment to retain a SIRMO improved patient satisfaction, masticatory ability with long-term survival rates [15, 16]. The ball and locator attachments are the most widely used attachments due to their convenient handling and reduced chair-side time. Ball attachment is even more popular because of its simplicity, cost efficiency [17], reduced denture mobility, and lower stresses transmitted to the underlying implants [18]. However, ball attachment cannot tolerate malalignments of implants, where rapid wear in its nylon retentive insert (female part, matrix) is observed with massive loss in its retention, especially in the first 3 months of denture wear [19]. A new attachment made from polyetherketoneketone (PEKK), which is a member of the polyaryletherketones (PAEKs), has been introduced. PAEKs have the advantage of high chemical and mechanical resistance to wear, besides its high tensile, fatigue, and flexural strength [20]. Moreover, it has been concluded that the combination of a titanium patrix and a matrix made from PEKK seems to be a promising combination for long-term retention when used with parallel and tilted implants [21–23].

Marginal bone loss (MBL) is an important biological indicator determining the prognosis of implant-supported/-retained overdentures. It has been reported that 1–1.5 mm of MBL during the first year of function and less than 0.2 mm annually is a criterion for implant success [24, 25]. Alsabeeha et al. 2011 [15] compared the MBL between the regular ball attachment, regular locator attachemnt and a large ball with wide implant and concluded that there was no statistically significant differences between them and that the regular ball and locator attachment performed similarily with acceptable MBL after 1-year follow-up. Coutinho et al. 2022 [26] concluded minimum MBL of 1.46 mm after a 5-year follow-up using a ball attachment for a SIRMO, which comes in agreement with Cordioli et al. 1997 [2] that concluded a minimum mean MBL of 1.42 ± 0.56 mm after 5-year follow-up using a ball attachment. Liddelow el al 2007 & 2010 [27, 28] reported that immediate loading using a SIRMO using a ball attachment resulted in physiologically acceptable MBL.

Studies comparing MBL in SIRMOs are, however, scarce. Hence, the aim of this randomized clinical trial (RCT) was to compare MBL of SIRMOs retained by ball and CM-LOC attachment after 2 years in clinical service, while using the submerged (S) and non-submerged (N) healing protocols. The null hypothesis is that there is no difference in MBL for the ball and CM-LOC attachemnts while using the Submerged (S) and Non-submerged (N) for the single implant-retained mandibular overdenture after 2-year follow-up.

## Materials and methods

The ethical committee of the Faculty of Dentistry, Cairo University, Egypt approved the study on June, 2016 (Ethical Approval Number: 16/6/10). The protocol of the current study was prepared in accordance with the SPIRIT statement for reporting clinical trials [29] and performed according to the Good Clinical Practice (GCP) and to the principles of the Declaration of Helsinki (2008). Moreover, the study was registered at the PAN AFRICAN CLINICAL TRIALS REGISTRY (Trial number: PACTR20183003085193). https://pactr.samrc.ac.za/TrialDisplay.aspx?TrialID=3085.

### Sample size calculation and randominization

Based on an RCT [15], the control group (ball attachment) showed MBL of 0.2 ± 0.4 mm after 1-year follow-up, while an estimated mean difference of 0.3 mm was reported for the locator group. To reject the null hypothesis, while using a power of 80%, a *p* < 0.05 and an independent t-test for comparing the MBL in ball and CM-LOC, 58 patients were needed (29 patients per group). Considering a dropout rate of 15% after 2 years, a total of 66 patients should be required; 33 in each group.

Eighty patients were recruited from the outpatient clinic of the Prosthodontic Department, Faculty of Dentistry, Cairo University, following strict inclusion criteria [30]. Patients were randomized by IR using sequentially numbered opaque sealed envelopes (SNOSE) at two stages of this RCT; after installing the implants and at time of attachment pick up in the denture. In the first stage they were randomized into submerged (S) and non-submerged (N) healing, and in the second stage, the S and N groups were further rerandomized using stratified randomization into ball (B) (Zimmer dental implants, Indiana, USA) and CM-LOC (C) (Cendres and Metaux, CM-LOC, SA, Biel, Switzerland) groups. Since blinding in the trial was not possible, randomization was carried out after installing the implants to decrease the risk of bias. All clinical steps and outcome assessment were carried out at Dr. Zekry’s clinic, in the Prosthodontic Department, Faculty of Dentistry, Cairo University. All patients had to fulfill the following criteria:

### Inclusion criteria

Completely edentulous patients of an age ranging from 50–69 years old were considered eligible. All patients should have a glycosylated haemoglobin (HbA1c) value ≤ 8 [31]. A Cone beam computed tomography (CBCT) should show bone width of ≥ 5 mm in the anterior region of the edentulous mandible. Patients should be of Class II or Class III candidate according to Mc Garry’s classification,1999 [32]. Patients who had no previous complete dentures, or those who had old technically acceptable mandibular dentures with unsatisfactory retention and accepted to improve the latter’s by installing a single midline implant, were included. For patients who received new dentures, a denture adaptation period of 6 weeks was considered. A Written informed consent was signed by all patients before implant placement.

### Exclusion criteria

Patients, who had an implant stability less than 60 ISQ, and insertion torque less than 30 N/Cm, showed allergic reactions to titanium, were non-compliant or decided to withdraw, were all excluded.

## Interventions

**Fig. 1:**
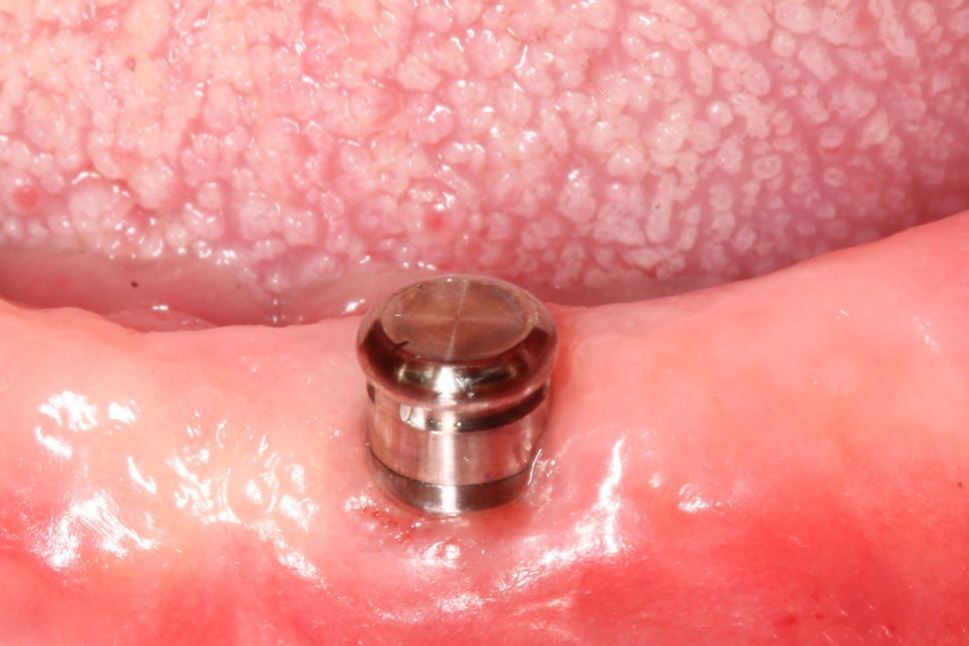
CM-LOC attachment

**Fig. 2:**
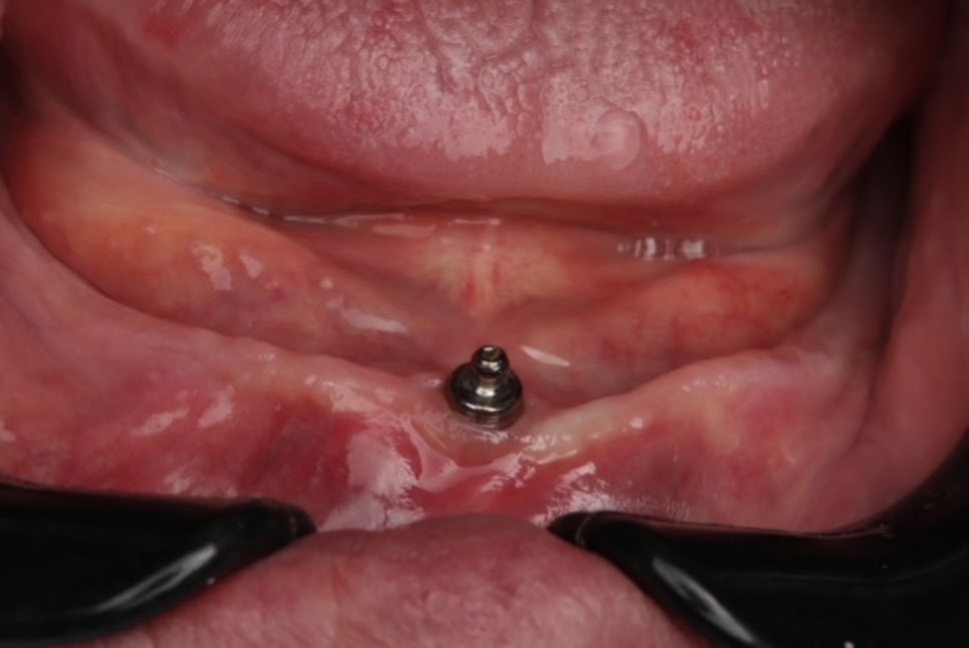
Ball attachment

A small crestal incision was made in the mandibular central incisor area. Sequential implant bed drilling was carried out following the manufacturer’s instructions. After installing the selected implant (Tapered Screw-Vent, Zimmer Biomet, Carlsbad, CA, USA) of a standardized size (3.7 × 10 mm), implant stability was measured using a resonance frequency analyzing device (Osstell, Integration Diagnostics Ltd., Sävedalen, Sweden) and a torque wrench to exclude implants with ISQ values < 60 and insertion torque < 30 Ncm. Based on the randomization, patients were assigned into N or S groups. In the N group patients received a healing abutment, while in the S group a cover screw was attached to the implant. Implants were left to heal for a 3 month-period, during which the patient was allowed to use a properly relieved and soft lined denture (GC soft liner, Corporation, Tokyo, Japan). At that stage patients were rerandomized based on the attachment system into group C (CM-LOC attachment) and B (Ball attachment) (Figs. 1, 2). In group C, the “medium’ (green) retentive inserts, while in group B the transparent nylon retentive inserts were used. The ball attachments had cuff heights of 2 and 4 mm, while the C attachments had 2-, 3-, 4- and 5-mm cuff heights. The required cuff height of the attachment was determined by a periodontal probe that measured the distance from the base of the implant to the upper border of the soft tissue.

The Recommended torque for screwing all attachments was 30 Ncm. The mandibular denture was then relieved opposite to the attachment which was picked up in the denture using a prepared mix of dual cured resin (LuxaTemp, DMG Dental, Hamburg, Germany). Patients were recalled to check for premature contacts and/or pressure areas.

**Fig. 3:**
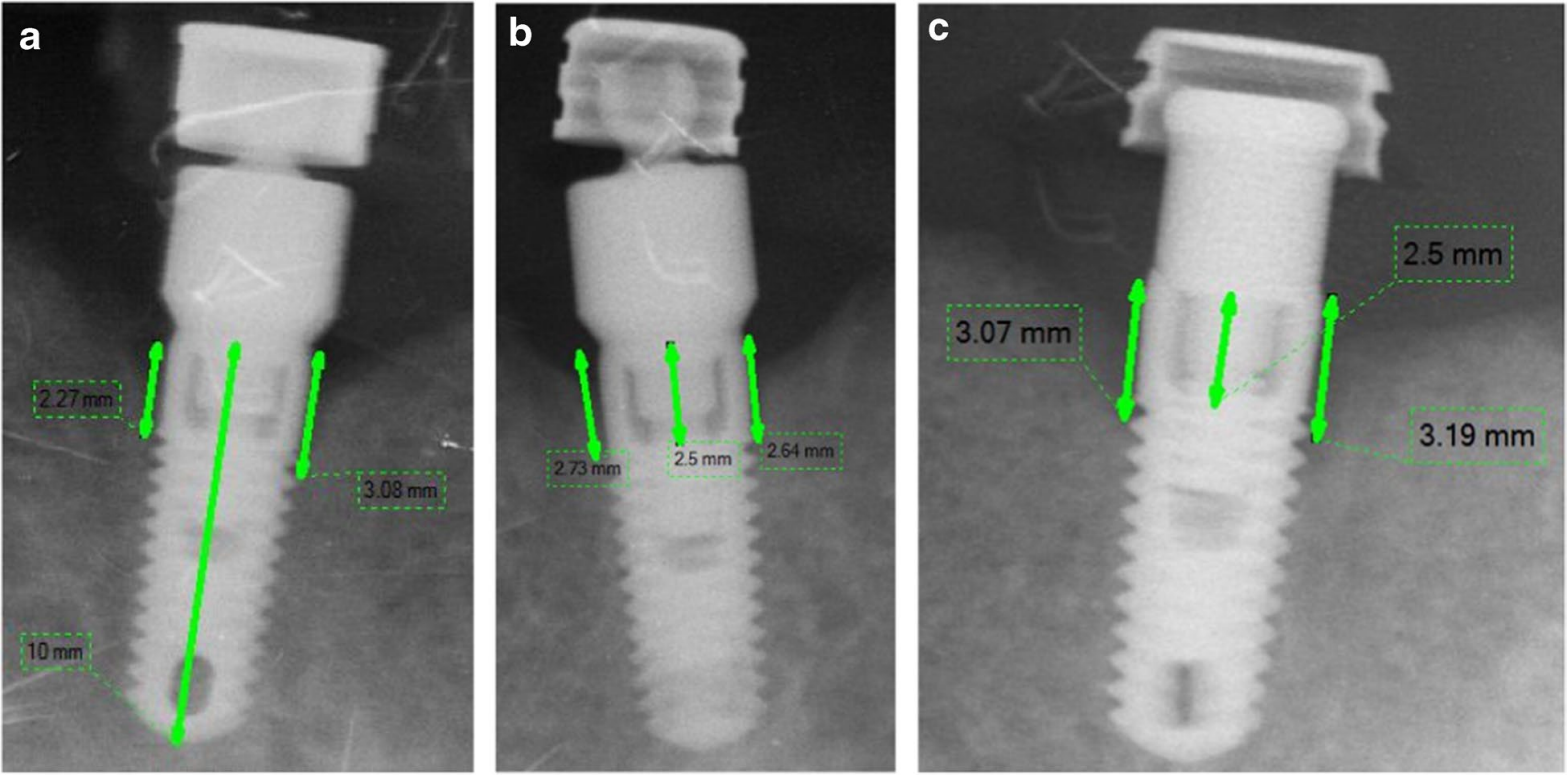
(**a**, **b** & **c**) showing marginal bone height changes

Long cone paralleling technique with standardized digital peri-apical radiographs using XCP Rinn bite block (Rinn XCP manufactures C. Ligin, III, USA) were employed to radiograph the patients at base line (day of pick-up), after 12 and 24 months. A viewing software (DIGORA® for windows—dfw 2.7 software, Soredex, Tuusula, Finland) was used for measuring the marginal bone height at the right and left sides of the installed implants. Image calibration was done in reference to implant length as well as to the distance between the implant shoulder and the first thread, which is in TSV Screw-Vent Zimmer company 2.5 mm (Fig. 3a, b). A horizontal line, coinciding with the implant platform level, was used as a reference line (RL). MBL was measured by measuring the height of a vertical line that lies perpendicular to RL and extends from it to the first bone-implant thread contact at the right and left sides (Fig. 3a, b, c). The same measurements were made by SN twice in a 2-week time interval.

### Statistical analysis

Data were statistically described in terms of mean ± standard deviation (± SD), or median and range accordingly. Numerical data were tested for normality using the Kolmogorov–Smirnov test. Comparison between the study groups was done using Mann Whitney *U* test for independent samples when comparing 2 groups and Kruskal Wallis test when comparing > 2 groups. Comparison over time was done using Wilcoxon Signed Rank test after applying Bonferroni adjustment of multiple comparisons. For binary or categorical data, chi square test was employed. Two-sided *p* < *0.05* was considered statistically significant. Intra-observer reliability was assessed using interclass correlation (ICC) coefficient with 95% confidence interval (CI). IBM SPSS (Statistical Package for the Social Science; IBM Corp, Armonk, NY, USA) release 22 for Microsoft Windows was used for all statistical analyses.

## Results

**Fig. 4:**
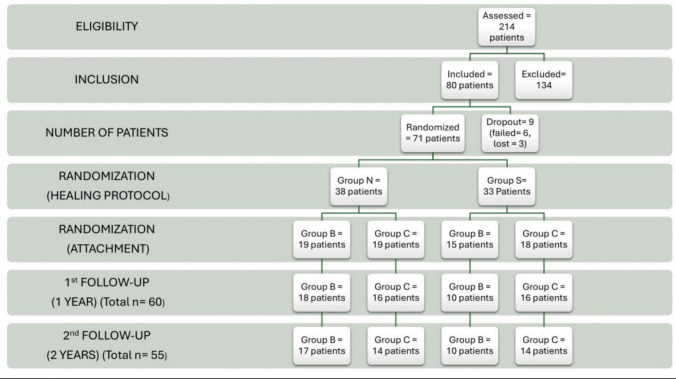
Participant flowchart

Two hundred and fourteen patients were screened, 134 of which did not fulfill the eligibility criteria and were therefore excluded. Finally, 80 patients were included in this RCT; 40 received ball (group B) and 40 CM-LOC attachment (group C). Included patients were 56 males and 23 females, with a mean age of 62.5 years for males and 59.6 years for females. At the end of the healing phase, 6 patients had implant failure (two from N group and 4 from S group). Moreover, three patients from S group were lost to follow-up before the pick-up appointment. A total of 71 patients received attachments as dictated by the randomization of the second stage (Fig. 4). After 2 years of follow-up, 27 patients in group B, and 28 patients in group C had been maintained. Taking a look at the baseline characteristics as shown in Table 1, reveals that all the studied variables (age, sex, collar height) were distributed between the studied groups with no significant differences except for the age distribution between groups S and N in the B group, where the age in the submerged healing protocol of this group was statistically higher (62.6 ± 4 versus 57.7 ± 5 years).

**Table 1:** Baseline characteristics of participants in the assigned groups and subgroups after 3 months of healing using X^2^ test for binary and Student’s t test for numerical data

Time point	Variable		N		S		*p*-value
			Mean ± SD	n	Mean ± SD	n	0.06
Before allocation to attachment	Age		60.4 ± 5	38	62.7 ± 4.6	33	
	Sex	M/F	–	22/16	–	24/9	0.192
After attachment allocation	Age	B	57.7 ± 5	19	62.6 ± 4	15	0.005*
		C	57.2 ± 4	19	55.9 ± 5.4	18	0.409
	Sex (M/F)	B	–	11/8	–	10/5	0.867
		C	–	11/8	–	14/4	0.347
	Collar height	B (2 mm/4 mm)	–	10/9	–	7/8	0.730
		C 2 mm	–	1	–	1	0.994
		C 3 mm	–	8	–	8	
		C 4 mm	–	5	–	5	
		C 5 mm	–	5	–	4	

*N* non-submerged healing protocol, *S* submerged healing protocol, *B* ball attachment, *C* CM-Lock attachment

Studying the effect of intervention on MBL revealed no significant difference neither between B and C groups nor between N and S groups (Table 2). The C and the S groups, regardless of the other intervention, showed a slight greater MBL when compared to groups B and N, respectively throughout the follow-up periods at *p* < 0.05. The same insignificant results were obtained even after combining the effect of both attachment and healing protocol (Table 3) at *p* < 0.05. These results were obtained at all follow-up periods (0–1, 1–2 and 0–2 years) as shown in Tables 2 and 3. Table 3 shows that the least MBL was observed during the second year in BN group (0.01 ± 0.45 mm) and that the greatest bone loss was observed after 2 years in BS group (0.49 ± 0.48 mm).

**Table 2:** Effect of attachment type regardless of the healing protocol and effect of healing protocol regardless of the attachment type, on the bone height changes at the different study periods using Mann Whitney test

Time period (nB/nC)	Group B mean ± SD (*n* = 34)	Group C mean ± SD (*n* = 37)	*P*-value
0–1 y (28/32)	0.21 ± 0.38	0.23 ± 0.28	0.33
1–2 y (27/28)	0.08 ± 0.40	0.12 ± 0.35	0.62
0-2y (27/28)	0.28 ± 0.44	0.38 ± 0.46	0.3
(nS/nN)	Group N (mean ± SD) (*n* = 38)	Group S (mean ± SD) (*n *= 33)	*P*-value
0–1 y (34/26)	0.18 ± 0.35	0.28 ± 0.31	0.10
1–2 y (31/28)	0.08 ± 0.40	0.13 ± 0.34	0.36
0-2y (31/28)	0.27 ± 0.39	0.42 ± 0.51	0.62

*n* number, *nB* number of ball attachments, *nC* number of CM-Lock attachments, *nN* number of non-submerged healing protocol, *nS* number of submerged healing protocol

**Table 3:** Studying the combined effect of the different healing protocols and the attachment type on the bone height changes at the different study periods using Kruskal Wallis test

Time period	Intervention 1 (*n*)	mean ± SD	Intervention 2 (*n*)	mean ± SD	*P*-value
0–1 y	BN (18)	0.19 ± 0.43	BS (10)	0.24 ± 0.33	0.1
			CN (16)	0.16 ± 0.26	1
			CS (16)	0.31 ± 0.3	0.93
	BS (10)	0.24 ± 0.33	CN (16)	0.16 ± 0.26	0.98
			CS (16)	0.31 ± 0.3	0.1
	CN (16)	0.16 ± 0.26	CS (16)	0.31 ± 0.3	0.65
1–2 y	BN (17)	0.01 ± 0.45	BS (10)	0.24 ± 0.27	0.44
			CN (14)	0.2 ± 0.31	0.63
			CS (14)	0.04 ± 0.39	1
	BS (10)	0.24 ± 0.27	CN (14)	0.2 ± 0.31	1
			CS (14)	0.04 ± 0.39	0.7
	CN (14)	0.2 ± 0.31	CS (14)	0.04 ± 0.39	0.86
0–2 y	BN (17)	0.18 ± 0.39	BS (10)	0.49 ± 0.48	0.48
			CN (14)	0.39 ± 0.39	0.65
			CS (14)	0.37 ± 0.55	0.89
	BS (10)	0.49 ± 0.48	CN (14)	0.39 ± 0.39	0.1
			CS (14)	0.37 ± 0.55	0.1
	CN (14)	0.39 ± 0.39	CS (14)	0.37 ± 0.55	1

*N* non-submerged healing protocol, *S* submerged healing protocol, *B* ball attachment, *C* CM-Lock attachment

Studying the effect of collar height on MBL revealed no significant difference among the different collar heights, whether in group B or C at all study periods as shown in Table 4. The least MBL was found during the second year in the 3 mm collar height of the C group (0.03 ± 0.22 mm), which is the shortest height found among C at the time slot, followed by the 4 mm in the B group (0.08 ± 0.43 mm) as shown in Table 4.

**Table 4:** Effect of collar height on the bone height changes in B and C groups along the different study periods using Chi square test

Collar height	C *(n *= 28)			B (*n *= 27)		
	0–1 y	1–2 y	0–2 y	0–1 y	1–2 y	0–2 y
2mm	–––––––-	–––––––-	–––––––	0.28 ± 0.35	0.09 ± 0.41	0.37 ± 0.45
3mm	0.22 ± 0.13	0.03 ± 0.22	0.28 ± 0.24	–––––––-	–––––––-	–––––––-
4mm	0.31 ± 0.38	0.18 ± 0.53	0.54 ± 0.70	0.18 ± 0.43	0.08 ± 0.43	0.26 ± 0.46
5mm	0.14 ± 0.21	0.15 ± 0.28	0.29 ± 0.48	–––––––-	–––––––-	–––––––-
*P*-value	0.33	0.16	0.54	0.30	0.44	0.35

*B* ball attachment, *C* CM Lock attachment

Studying the effect of time on the MBL revealed significant loss in the first year of the follow-up and after 2 years in group B (0-1y = 0.21 ± 0.38 mm, *p* = 0.018; 0-2y = 0.27 ± 0.39 mm, *p* = 0.012) and C (0-1y = 0.23 ± 0.28 mm, *p* = 0.000; 0-2y = 0.38 ± 0.46 mm, *p* = 0.003) regardless of the healing protocol, and in group CS in the first year (0-1y = 0.31 ± 0.30 mm, *p* = 0.006), and in group BS after 2 years (0-2y = 0.49 ± 0.48 mm, *p* = 0.021) (Table 5).

**Table 5:** Effect of time on the bone height changes in B and C groups when considering the healing protocol one time and when disregarding it the other time using Wilcoxon signed rank test with Bonferroni adjustments for multiple comparisons

Intervention	n0/n1	0–1 y	n1/n2	1–2 y	n0/n2	0–2 y
B	34/28	0.21 ± 0.38	28/27	0.08 ± 0.40	34/27	0.27 ± 0.39
*P*-value		0.018*		0.12		0.012*
C	37/32	0.23 ± 0.28	32/28	0.12 ± 0.35	37/28	0.38 ± 0.46
*P*-value		0.000*		0.15		0.003*
BN	19/18	0.19 ± 0.43	18/17	0.01 ± 0.45	19/17	0.18 ± 0.39
*P*-value		0.098		0.96		0.140
BS	15/10	0.24 ± 0.33	10/10	0.24 ± 0.27	15/10	0.49 ± 0.48
*P*-value		0.07		0.31		0.021*
CN	19/16	0.16 ± 0.26	16/14	0.2 ± 0.31	19/14	0.39 ± 0.39
*P*-value		0.08		0.14		0.07
CS	18/16	0.31 ± 0.3	16/14	0.04 ± 0.39	18/14	0.37 ± 0.55
*P*-value		0.006*		0.35		0.07

*N* non-submerged healing protocol, *S* submerged healing protocol, *B* ball attachment, *C* CM-Lock attachment

The intra-class correlation coefficient of the average values at the baseline, after 1 year, and after 2 years showed a high statistically significant reliability (ICC = 0.991; ICC = 0.999; ICC = 0.997, *p* = 0.000).

## Discussion

The criteria of implant success have been reported first by Albrektsson and colleagues in 1986 [33], and then later by many authors [28, 34–38]. All agreed that MBL up to 1.5 mm during the first year of function might indicate successful osseointegration followed by a maximum of 0.2 mm annually. Results of the present trial has failed to reject the null hypothesis, as both attachments used; ball and CM-LOC showed a physiologically acceptable MBL during the first year of function: 0.213 ± 0.3814 mm for B, and 0.230 ± 0.2823 mm for C group. This comes in agreement with the study of Alsabeeha et al., that reported MBL of 0.2 ± 0.4 mm for ball attachment and 0.23 ± 0.44 mm for a Locator attachment, after 1 year of clinical service [15]. After 2 years, group B showed 0.279 ± 0.4411 mm MBL, while C group had a slightly higher MBL (0.380 ± 0.4624 mm), with no statistically significant difference between them. An explanation for the slight difference between them is that the nylon matrix of the ball attachment does not allow for any vertical resiliency during mastication with fewer denture base rotation [39]. Unlikely, the CM-LOC attachment has a slot in its PEKK matrix design that does not engage the central hole as in the locator attachment and that expands during loading, resulting in greater vertical resiliency, thereby allowing for greater denture base rotation [40]. Besides, PEKK is an elastic biomaterial with good shock absorbtion, thereby would decrease the bone loss around the implants [41]. However, rotational movement around the attachment is highly expected in a SIRMO prosthesis. CM-LOC might have some pivoting action in response to this movement, on contrary to the ball attachment, which has a neck that absorbs greater energy, and hence reduces the stresses and MBL around the implants [42, 43]. This might explain the insignificant difference between them with the slightly greater MBL observed in CM-LOC attachment.

Selection of the cuff height of the attachments used in this study was greatly dependent on the thickness of the surrounding soft tissues. However, in both attachments the cuff height had an insignificant effect on the MBL. Generally speaking, an increase in the attachment height creates greater nonaxial bending forces, due to the increase in the vertical cantilever effect, which consequently increases the stresses transferred to the implant and might lead to increased MBL. This might explain why the CM-LOC attachment, 3 mm in height, showed the least marginal bone height changes compared to the 4- and 5-mm heights. While, in the ball attachment the opposite was observed. MBL was greater in the 2 mm than in the 4 mm height. This could be attributed to the different mechanism of action of the ball attachment. The higher collar of the ball attachment is associated with a wider base, that tends to distribute the stresses better on the implants and hence leading to less MBL in the 4 mm than in the 2 mm cuff heights [44].

Several studies revealed the success and clinical viability of SIRMO [21, 45–48]. Padmanabhan et al. concluded in their systematic review that SIRMO is a cost-effective, minimally invasive treatment modality that restores function to old edentulous patients with high implant success rates and minimal complications [49]. Very few studies have compared submerged and non submerged healing protocol concerning the SIRMO treatment option. The aim of the present trial was to compare the MBL of both attachments, while using the two different healing protocols; S and N for completely edentulous patients.

Regarding the effect of the healing protocol, on the MBL changes, an insignificant difference was found between the S and N groups, slightly favoring the N group. It was reported that early functional loading might prevent marginal bone resorption and leads to successful osseointegration [50, 51]. In addition, it was concluded that new bone formed under loading conditions is mainly made out of mature lamellar bone that has a greater density when compared to the newer bone formed under unloaded conditions [52, 53]. This phenomenon is referred to as “form follows function” and might explain why the N in both attachments showed lesser MBL compared to S group during the first year. This might also explain why the effect of time on the MBL became statistically significant in the S group of both attachments, B after 2 years and C during the first year. The only difference here lies in the timing of the significant difference appearance, where most of the loss took place in the first year for the C, while the loss in the B group was more evenly distributed among the 2 years. This could be attributed to the design differences that were explained above. Disregarding the healing protocol, there was a significant bone loss for both attachments after 2 years and that could be attributed to the physiologic bone remodeling that was associated with the implant loading. However, and as previously explained none exceeded the physiological limit for bone loss neither during the first, nor during the second year.

All patients in the present randomized trial were completely edentulous, as the concept of the single implant retained mandibular overdenture is only indicated for completely edentulous patients. A systematic review carried out by Zhou et al. 2023 concluded that restoration of the edentulous arch by implants would significantly improve the bitting force and chewing efficiency compared with conventional dentures[54]. So it is expected that if the opposing arch in the present trial was a full arch prosthesis opposed by a SIRMO the marginal bone loss would be expected to be greater than those reported by our study and of similar studies with opposed complete denture due to the greater forces that would be directed to the mandibular single implant. Very smilarly if the opposing arch is a full fixed natural dentition, the forces directed would be of greater magnitude than that of complete denture which could result in overloading of the SIRMO. So it is really worthnoting that SIRMO would be a very reliable treatment modality for rehabilitation only for compleletly edentulous patients.

Age is an important factor for bone maintenance, as it is known that bone mass density decreases with aging, i.e. age-related osteoporosis in male and female patients. It is also known that age-related bone loss is predominant in the cancellous bone. Negri et al. concluded that age could be an important factor that would affect marginal bone height changes [55], in agreement with this study it was revealed in the present study that the group BS that showed the highest significant MBL (0.49 ± 0.48 *p* = 0.021) after a 2 years follow-up period. It was also the group that showed a significant increase in age group (62.4 ± 4, *p* = 0,005) (Table 1). Therefore, age could be a reason for the increased MBL for the BS group.

The limitations present in the following trial was that the number of males were greater than that of females. Moreover, there was significant difference in the age between the S and N groups. But that was due to the randomization that was carried out regardless of the gender. Furthermore, the number of patient drop-outs in the first year and second year were only 5 patients, thus maintaining 55 patients in the second year, which is considered to be a point of strength in the current trial, could add to the reliability of the results.

## Conclusion

Both attachments showed accepted limits of marginal bone loss, with the non-submerged healing protocol performing better after a 2-year follow-up period.

## Data Availability

No datasets were generated or analysed during the current study.
